# Supplementary material for: Understanding the use of patient-reported data by health care insurers: A scoping review
Source: PLoS One. 2020 Dec 28;15(12):e0244546. doi: 10.1371/journal.pone.0244546 (PMC7769438; doi:10.1371/journal.pone.0244546)
Supplement: S2 File — (PDF) [file pone.0244546.s002.pdf]

S2 File. Full search strategy for each database.

PUBMED
("health insurance"[Title/Abstract] OR "health insurer"[Title/Abstract] OR "private health insurance"[Title/Abstract] OR "private health insurer"[Title/Abstract] OR "payer"[Title/Abstract] OR "purchaser"[Title/Abstract] OR "medical insurance"[Title/Abstract] OR "medical care insurance"[Title/Abstract] OR "private health plans"[Title/Abstract] OR "third party payer"[Title/Abstract] OR "national health insurance"[Title/Abstract] OR "social health insurance"[Title/Abstract] OR "co-insurance"[Title/Abstract] OR "group health insurance"[Title/Abstract] OR "statutory health insurance"[Title/Abstract] OR "statutory healthcare insurance"[Title/Abstract]) AND ("Patient-reported data"[Title/Abstract] OR "patient reported"[Title/Abstract] OR "patient reported experience"[Title/Abstract] OR "PREM"[Title/Abstract] OR "PREMS"[Title/Abstract] OR "PROM"[Title/Abstract] OR "PROMS"[Title/Abstract] OR "Patient-reported experiences"[Title/Abstract] OR "patient-reported outcomes"[Title/Abstract] OR "quality indicators"[Title/Abstract] OR "performance indicators"[Title/Abstract] OR "patient reported feedback"[Title/Abstract] OR "Patient satisfaction"[Title/Abstract] OR "consumer satisfaction"[Title/Abstract] OR "patient questionnaire"[Title/Abstract] OR "consumer reported data"[Title/Abstract] OR "consumer experience"[Title/Abstract] OR "consumer questionnaire"[Title/Abstract] OR "consumer survey"[Title/Abstract] OR "patient survey"[Title/Abstract] OR "consumer need"[Title/Abstract] OR "consumer expectations"[Title/Abstract] OR "consumer preferences"[Title/Abstract] OR "patient need"[Title/Abstract] OR "patient expectation"[Title/Abstract] OR "patient preference"[Title/Abstract] OR "consumer feedback"[Title/Abstract])

EMBASE
((health insurance or health insurer or private health insurance or private health insurer or payer or purchaser or medical insurance or medical care insurance or private health plans or third party payer or national health insurance or social health insurance or co-insurance or group health insurance or statutory health insurance or statutory healthcare insurance) and (Patient-reported data or patient reported or patient reported experience or PREM or PREMS or PROM or PROMS or Patient-reported experiences or patient-reported outcomes or quality indicators or performance indicators or patient reported feedback or Patient satisfaction or consumer satisfaction or patient questionnaire or consumer reported data or consumer experience or consumer questionnaire or consumer survey or patient survey or consumer need or consumer expectations or consumer preferences or patient need or patient expectation or patient preference or consumer feedback)).ab.

HEALTH SYSTEM EVIDENCE DATABASE
"health insurance" OR "health insurer" OR "payer" OR "purchaser" OR "medical insurance" AND "Patient-reported data" OR "patient reported" OR "patient reported experience" OR "patient need" OR "patient expectations" OR "patient preference" OR "consumer preference" OR "consumer experience" OR "consumer need"

NICE
"health insurance" OR "health insurer" OR "payer" OR "purchaser" AND "Patient reported data" OR "patient reported" OR "patient reported experience"

JSTOR
(ab:(health insurance) OR (payer) OR (purchaser) OR (third party payer) OR (insurer) AND ab:(consumer experience) OR (consumer satisfaction) OR (consumer reported outcomes))

EMERALD
"health insurance" OR "health insurer" OR "payer" OR "purchaser" OR "medical insurance" AND "Patient-reported data" OR "patient reported" OR "patient reported experience" OR "patient need" OR "patient expectations" OR "patient preference" OR "consumer preference" OR "consumer experience" OR "consumer need"

Wiley Online Database
Search 1 on the 26th of May 2019
“"health insurance" OR "health insurer" OR insurer OR insurance" anywhere and ""consumer preferences" OR "consumer expectation" OR "consumer satisfaction" OR "consumer experience" OR "consumer reported data" OR "patient satisfaction" OR "patient report*" OR "patient-reported data" OR "patient experience" OR "patient reported experience" OR "patient reported outcome" OR "PREM" OR "PROM"" in Keywords

Search 2 on the 26th of May 2019
“"health insurance" OR "health insurer" OR insurer OR insurance" Title and ""consumer preferences" OR "consumer expectation" OR "consumer satisfaction" OR "consumer experience" OR "consumer reported data" OR "patient satisfaction" OR "patient report*" OR "patient-reported data" OR "patient experience" OR "patient reported experience" OR "patient reported outcome" OR "PREM" OR "PROM"" in Keywords

Search 3 on the 26th of Math 2019
“"health insurance" OR "health insurer" OR insurer OR insurance" Keywords and ""consumer preferences" OR "consumer expectation" OR "consumer satisfaction" OR "consumer experience" OR "consumer reported data" OR "patient satisfaction" OR "patient report*" OR "patient-reported data" OR "patient experience" OR "patient reported experience" OR "patient reported outcome" OR "PREM" OR "PROM"" in Keywords

Cochrane Library/Cochrane Collection Plus
Search on the 21th of May
“health insurers” and “patient reported data”, “PREM”, “PROM”

PDQ-Evidence for informed Health Policymaking
Search on the 26th of May
“health insurers” and “patient reported data”, “PREM”, “PROM”

NIHR – Involve
Search on the 27th of May
“health insurers” and “patient reported data”, “PREM”, “PROM”

Health Business Elite
Search on the 27th of May
“health insurers” and “patient reported data”, “PREM”, “PROM”
