# Supplementary material for: Understanding the use of patient-reported data by health care insurers: A scoping review
Source: PLoS One. 2020 Dec 28;15(12):e0244546. doi: 10.1371/journal.pone.0244546 (PMC7769438; doi:10.1371/journal.pone.0244546)
Supplement: S3 File — (PDF) [file pone.0244546.s003.pdf]

S3 File. Overview of the organizations whose websites were consulted during the non-systematic search.

- **Websites of health insurers**
  - Aetna
  - AOK
  - AXA global
  - Barmer
  - BKK
  - Blue Cross US
  - BUPA
  - DAK
  - Generali
  - HCF private health insurance
  - IBM Japan health insurance
  - Kaufmännische Krankenkasse
  - Knappschaft
  - Medibank
  - Private healthcare Australia
  - Southern Cross Health Society
  - Swedish social insurance agency
  - Techniker Krankenkasse
- **Insurance associations/federations**
  - Association International de la Mutualité
  - Canadian Life and Health insurance association
  - European (re) insurance federation
  - European Alliance of social insurances
  - European Health Management Association
  - French insurance federation
  - Global Federation of Insurance Associations
  - International federation of health plans
  - International Social Security association
- **Consultancy firms**
  - Avisa
  - Bruegel
  - Brunswick group
  - Ecorys
  - Edelmann
  - Ernest and Young International
  - Health Consumer Powerhouse
  - KPMG
  - LaingBuisson
  - McKinsey

- **Key policy-making organizations**
  - European Commission
  - European Observatory on health systems and policy
  - Organisation for Economic Co-operation and Development
  - RAND
  - World Health Organization
    - Policy briefs and summaries
    - Europe’s Health Evidence Network
- **European Organizations and Projects**
  - Actuarial Association Europe
  - European Centre for International Political Economy
  - European Collaboration for health Optimization
  - European Consumer Organization
  - European Health Economics Associations
  - European Hospital and Healthcare Federation
  - European Institute of innovation and technology in health
  - European Patient Academy
  - European Public Health Association
  - European Union Network for Patient Safety and Quality of Care, PaSQ joint Action
  - Geneva associations
  - Quality improvements in hospitals across Europe (DUQuE project)
- **Miscellaneous**
  - Alliance for health care competitiveness
  - Canadian Institute for Health Information (CIHI)
  - Care Quality Commission
  - Corporate Europe
  - Deutsche Arbeitsgemeinschaft Selbsthilfegruppen
  - Deutsche Institute für Medizinische Dokumentation und Information
  - DICA
  - Federal ministry of health
  - G-BA
  - Health Experience Research Group (Nuffield Department of Primary Care)
  - Healthtalks.org
  - ICHOM
  - IGES
  - Institute für Qualitätssicherung und Transparenz im Gesundheitswesen
  - Kings Fund
  - Krankheitserfahrungen.de
  - London School of Economics and Political Science
  - Medizinischer Dienst der Krankenkassen
  - Optimedis
  - PatientsLikeMe
  - Picker Institute
  - Robert Koch Institute
  - Scuola Superiori di Studi Universitari e di Perfezionamento Sanr’Anna

- Spitzenverbände der Gesetzlichen Krankenversicherer
- The Office of the National Coordinator for health information Technology – USA
- The Royal College of General Practitioners and Surveillance Centre
- University of Toronto
- Verband der privaten Krankenversicherer
- Verbraucherzentrale
- Zorginzicht
